# Trimethoprim/Sulfamethoxazole‐Induced Systemic Toxic Epidermal Necrolysis Syndrome: A Case Report

**DOI:** 10.1002/ccr3.72512

**Published:** 2026-04-23

**Authors:** Qing Wang, Litong Chen, Wen Ye

**Affiliations:** ^1^ Department of Critical Care Medicine Anhui Provincial Corps Hospital of the Chinese People's Armed Police Force Hefei Anhui Province China; ^2^ Department of Gastroenterology Huangpu People's Hospital of Zhongshan City Zhongshan China

**Keywords:** cutaneous adverse reaction, rare disease, skin lesion, toxic epidermal necrolysis, trimethoprim/sulfamethoxazole

## Abstract

Trimethoprim/sulfamethoxazole can induce life‐threatening toxic epidermal necrolysis. Early recognition, immediate drug cessation, and multidisciplinary supportive care are critical. Even with aggressive therapy, mortality remains high, and SCORTEN score aids in prognostication.

## 1 Introduction

Toxic epidermal necrolysis is a severe and rare life‐threatening dermatologic condition characterized by extensive epidermal necrosis and detachment, often involving large areas of the body surface. With an estimated annual incidence of 2–7 cases per million population, it represents a rare but devastating cutaneous adverse reaction [1, 2]. Drugs are the most common cause of TEN, usually causing disease in adults within 8 weeks. Among them, anticonvulsants, antimicrobial sulfonamides, allopurinol, and anticonvulsants are common [3]. We herein report a particularly severe case of TEN, involving > 90% of the body surface area, which developed following treatment with the sulfonamide antibiotic trimethoprim/sulfamethoxazole (TMP‐SMZ).

## 2 Case Presentation/Examination

**FIGURE 1:**
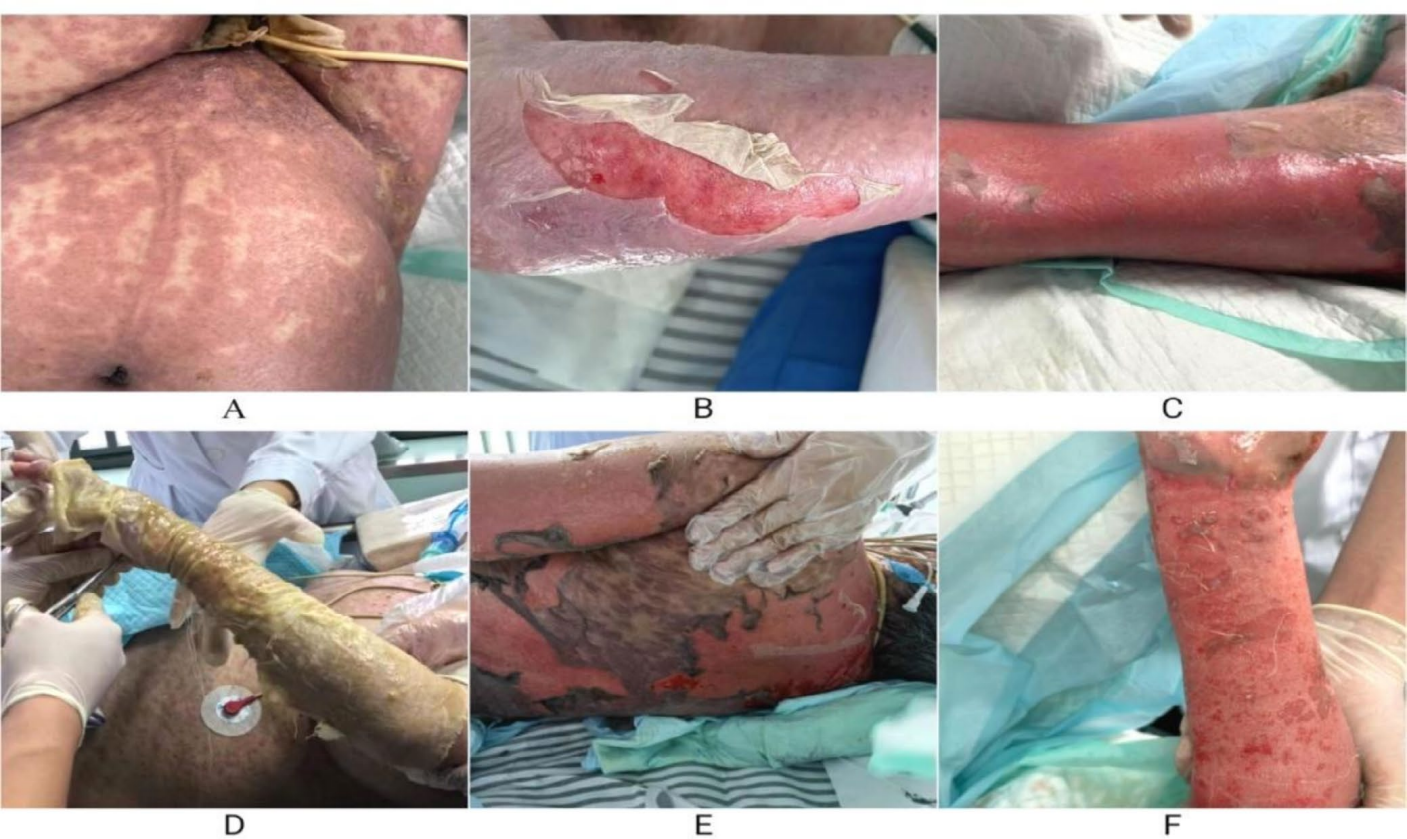
(A) The patient developed a large generalized red rash, partially fused into patches, one week after treatment with TMP‐SMZ. (B, C) The patient had multiple blisters all over the body that ruptured and peeled off the skin. (D) Wrap the patient's limb with a sterile dressing. (E, F) Partial skin regeneration was observed in the patient.

This is a 58‐year‐old woman who was unconscious for more than 10 months after cardiopulmonary resuscitation. After admission, a sputum culture examination suggested *Acinetobacter baumannii* , and the use of TMP‐SMZ was selected according to the drug sensitivity test results. One week after receiving TMP‐SMZ treatment, the patient began to develop dense red pimples partially fused into patches (Figure 1A), and her body temperature rose to 39.4°C. Laboratory findings suggest inflammation with a neutrophil percentage of 83.7%; C‐reactive protein was 54.75 mg/L, procalcitonin was 3.75 ng/mL. We examined the patient for anti‐nuclear antibodies (ANA), anti‐dsDNA, anti‐neutrophil cytoplasmic antibodies (ANCA), and rheumatoid arthritis‐related blood tests, and these proved to be negative (Table 1).

**TABLE 1:** Laboratory test results for the patient.

Marker	Level^a^	Level^b^	Reference
White blood cells	8.36	2.33	3.5–9.5 × 10^9^/L
Neutrophils	83.7	78.8	50%–70%
CRP	54.75	137.98	0–10 mg/L
Procalcitonin	3.75	2.81	< 0.05 ng/mL
Hemoglobin	112	113	115–150 g/L
Alanine aminotransferase	49	58	0–40 U/L
Aspartate aminotransferase	41	65	0–35 U/L
Albumin	31.2	35.9	35–55 g/L
Creatinine	61	39	41–81 μmol/L
Blood urea nitrogen	9.1	9.5	2.6–7.5 mmol/L
PT	15.1	14.9	10–14.5S
APTT	44.6	43.2	28–43S
Rheumatoid factor	15	—	0–20 IU/mL
ANCA	Negative	—	Negative
Anti‐dsDNA	Negative	—	Negative
Antineutrophil antibodies	Negative	—	Negative
Hepatitis B surface antigen	Negative	—	Negative
HIV antibodies	Negative	—	Negative
Blood cultures	—	Negative	Negative
Skin wound secretion culture	—	Negative	Negative

Abbreviations: APTT, activated partial thromboplastin time; CRP, C‐reactive protein; PT, prothrombin time.

^a^ Initial laboratory data of the patient.

^b^ Laboratory data for patient review.

## 3 Methods (Differential Diagnosis, Investigations and Treatment)

We administered loratadine tablets nasally, hydrocortisone intravenously, and immediately discontinued TMP‐SMZ treatment, but over the next few days the patient's rash expanded and evolved into a large, generalized erythematous rash and blisters, followed by rupture of the blisters, skin breakdown and exfoliation(Figure 1B,C). The patient's generalized skin lesions continued to develop, with a rash involving the face, chest, abdomen, perineum, anterior thighs, ankles, shoulders, and upper arms, as well as the mucous membranes of the eyes, mouth, and pharynx, which covered approximately 90% or more of her Body Surface Area (BSA) (using Wallace's Rule of Nine). We rechecked the parameters and the percentage of neutrophils was 78.8%, and the C‐reactive protein was 137.98 mg/L. Meanwhile, the results of the pathogenetic tests showed that the blood cultures and the cultures of the secretions of the skin trauma were negative, and the CT of the chest suggested only a little inflammation of the lower lungs bilaterally and pleural effusions, which were roughly similar to those at the time of the admission to the hospital. Evaluation by the Naranjo scale showed a probable causal association of the drug with adverse reaction, and the only treatment involved was TMP‐SMZ in the days before the development of the skin lesions. Based on the patient's history and typical clinical presentation, the diagnosis of TEN was made with a SCORTEN score of 5. The next treatment consisted of intravenous human immunoglobulin 400 mg/kg/day, intravenous dexamethasone 20 mg/day, application of human epidermal growth factor to promote wound healing and wrapping of sterile dressings around the severe areas (Figure 1D). Over the next week, partial skin regeneration (Figure 1E,F) and improvement in inflammatory markers were observed in the patient, but the patient developed severe renal failure and metabolic acidosis. The patient's family requested that the treatment be abandoned, and the patient was finally discharged from the hospital.

## 4 Conclusion and Results (Outcome and Follow‐Up)

We report a severe and ultimately fatal case of toxic epidermal necrolysis induced by trimethoprim/sulfamethoxazole in a critically ill patient. The diagnosis was clinically established based on the rapid progression of blistering and skin detachment affecting over 90% of the body surface area, significant mucosal involvement, and a high SCORTEN score of 5. Management involved immediate withdrawal of the culprit drug, combined immunomodulatory therapy (intravenous immunoglobulin and corticosteroids), and intensive supportive care. Although partial skin re‐epithelialization was achieved, the patient succumbed to complications including renal failure and metabolic acidosis. This case underscores the paramount importance of vigilance for severe cutaneous adverse reactions when prescribing high‐risk medications like TMP‐SMZ, particularly in vulnerable patients. It also highlights the utility of the SCORTEN score for risk stratification and the challenging reality that mortality remains substantial even with modern therapeutic approaches.

## 5 Discussion

Sulfonamides are a class of synthetic, broad‐spectrum antibacterial drugs that inhibit bacterial growth and replication by competing with the bacterial enzyme dihydrofolate synthase and by affecting bacterial nucleic acid synthesis. Currently, commonly used sulfonamide formulations include sulfadiazine, sulfamethoxazole, and combination formulations including salicylazosulfapyridine and trimethoprim/sulfamethoxazole (TMP‐SMZ, also known as cotrimoxazole). Hypersensitivity to sulfonamides occurs in approximately 1%–2% of patients and is preceded by preexisting symptoms such as fever, rash, facial edema, enlarged lymph nodes, arthralgia, and eosinophilia or atypical lymphocytosis. Injuries usually appear suddenly within 1–3 weeks of starting treatment. Hepatotoxicity of sulfonamides has been reported [4] as a possible cause of sulfonamide‐induced hypersensitivity reactions and has been associated with many cases of DRESS (Drug Rash with Eosinophilia and Systemic Symptoms), as well as Stevens Johnson Syndrome and Toxic Epidermal Necrolysis Syndrome.

Toxic epidermal necrolysis syndrome (TEN), also known as Leyll's syndrome, is a rare but extremely severe skin‐mucosal reaction characterized by extensive epidermolytic necrolysis, detachment, and vesicular blister formation. TEN is most often clinically induced by drugs, and its onset is rapid, with lesions rapidly expanding throughout the body, fusing with each other, followed by blisters or blood blisters and a positive Nikolsky's sign. It may be accompanied by multisystem involvement (respiratory and gastrointestinal mucosal damage, renal impairment, etc.).

Our patient developed TEN after first receiving TMP‐SMZ for a lung infection involving approximately her entire body surface area. TEN is a rare disease with a range of 2–7 cases per million reported annually. SJS, the milder form, is three times more common than TEN [5] and the results of a large study that included more than 20 million cases showed [6] that the SJS per million incidence rates were 8.61–9.69 for SJS, 1.46–1.84 for SJS/TEN, and 1.58–2.26 for TEN. Another large‐scale 1‐year follow‐up study of SJS/TEN patients showed [7] an increase in mortality over time, with 1‐year mortality rates of 24%, 43%, and 49% for SJS, SJS/TEN, and TEN, respectively.

Among the TEN cases reported so far, most of them are related to drug reactions. Common exogenous drugs that can induce TEN include anticonvulsants, antidepressants, sulfonamides, nonsteroidal anti‐inflammatory drugs, anti‐infective drugs, and targeted drugs that have been widely used in recent years. The exact mechanism of TEN is not fully understood, and an increasing number of studies suggest that the pathogenesis of TEN is related to cytotoxic T‐cell (CTL)‐mediated human leukocyte antigen (HLA)‐dependent drug hypersensitivity. Drugs and/or their active metabolites are presented by antigen‐presenting cells (APCs) and interact with HLA‐associated proteins, resulting in a strong enough signal from the T‐cell receptor (TCR) to activate the cells. Upon activation, stimulated CD^8+^ cytotoxic T cells release a range of cytokines or chemokines including perforin/granzyme, Fas‐FasL, TNF‐α, and granulysin, which may lead to the death of keratinocytes and mucosal cells, which may in turn lead to skin exfoliation and necrosis [8]. At the same time, CTL and NK cells infiltrate the skin to form blisters. NK and T cells generate granulysin, a proinflammatory molecule that induces cell death by disrupting target cell membranes [9, 10]. A prohapten model has now been proposed for sulfamethoxazole. Sulfamethoxazole, a sulfonamide antibiotic, exerts its antibacterial effect by inhibiting the bacterial enzyme dihydropteroate synthase, thereby disrupting folate synthesis and subsequent nucleic acid production [11]. This drug undergoes a rapid metabolic process and self‐oxidizes to nitro‐sulfamethoxazole (SMX‐NO), which possesses chemotactic properties and binds to intracellular proteins [12].

Before the onset of TEN, patients may experience nonspecific prodromal symptoms such as itchy skin, high fever, dyspnea, and rash. With the rapid progression of the disease, the lesions expand rapidly, blend into each other, and generalize throughout the body, with the upper trunk, proximal extremities, face, and mucosal areas (eyes, mouth, nose, genitals) usually being the first areas affected. The epidermis blisters under light pressure, the skin detaches, and when the dermis is exposed, large amounts of fluid are lost, which increases the risk of skin infection. Although the skin is most involved, multiple organ systems, such as the cardiovascular, pulmonary, gastrointestinal, and urinary systems, may also be affected. Severely ill patients exhibit a variety of complications, such as pneumonia, hepatitis, and sepsis, which are thought to be key contributors to increased morbidity and mortality [13]. The definitive diagnosis of TEN relies on histopathologic biopsy findings, but biopsy may prove to be an invasive and time‐consuming procedure. Clinical diagnosis is based on the characteristic lesions of the disease, predisposing factors, the area of mucosal involvement, and the patient's symptoms. If a patient may have TEN, appropriate therapeutic measures should be initiated as soon as possible, even if further histologic examination is required.

When a drug produces an adverse reaction, the first step is to discontinue it immediately. Based on the immunological mechanism of TEN, glucocorticoids, intravenous immunoglobulin (IVIG), cyclosporine, and TNF‐α antagonists are usually chosen as an overall therapeutic strategy for TEN in clinical practice. In addition, several techniques can be applied to the topical care of skin injuries; when a large number of large blisters are present on the skin, the blister fluid should be aspirated or released so that the tops of the blisters settle on the underlying dermis. During the acute phase of TEN, it is beneficial to apply a light oil emollient to the entire skin (including exposed areas) regularly to limit transepidermal water loss, improve barrier function, and induce re‐epithelialization of the skin [14]. The dermal exposure of TEN patients leads to an increased risk of infection and significant fluid loss, including loss of substances such as electrolytes and proteins. Therefore, patients with extensive skin defects should be treated with concomitant supportive therapy, including nutritional support, fluid resuscitation, and infection prevention.

Acute systemic damage can lead to multiple organ failure and even death in patients with TEN. Guidelines recommend the use of the SCORTEN scoring system for prognostic evaluation of patients with TEN, which consists of seven parameters: age, heart rate, serum urea level, presence or absence of malignancy, blood glucose level, area of epidermal desquamation, and bicarbonate level, with a score of 1 each, and with the increase in the cumulative score, the expected mortality rate of the patient increases [15]. In our case, the patient had a SCORTEN score of 5 and the predicted mortality was 85%.

### 5.1 Strengths and Limitations

The strength of this report lies in the comprehensive clinical, laboratory, and photographic documentation of a fulminant TEN case, providing a clear temporal link to TMP‐SMZ exposure. However, this study has limitations. First, it is a single‐case report, which inherently limits generalizability. Second, a confirmatory skin biopsy was not performed. The diagnosis was made clinically due to the highly characteristic presentation and the urgency of initiating treatment in an unstable patient. This clinical diagnosis is consistent with established practice when phenotypic features are classic. Third, while the Naranjo scale indicated a probable association, causality in a single case cannot be definitively proven. Finally, we have now reported this serious adverse drug reaction to the national pharmacovigilance database, as is crucial for ongoing drug safety monitoring.

## Author Contributions

**Qing Wang:** data curation, formal analysis, methodology, project administration, resources, software, validation, writing – original draft, writing – review and editing. **Wen Ye:** formal analysis, methodology, project administration, supervision, writing – review and editing. **Litong Chen:** conceptualization, formal analysis, investigation, supervision, writing – review and editing.

## Funding

The authors have nothing to report.

## Disclosure

This case highlights trimethoprim/sulfamethoxazole as a high‐risk trigger for toxic epidermal necrolysis. Early recognition, prompt drug withdrawal, and aggressive supportive care are essential. A SCORTEN score of 5 predicted high mortality, underscoring the need for vigilance even with immunomodulatory therapy.

## Ethics Statement

This study was approved by the Ethics Committee of the Armed Police Anhui Provincial General Hospital.

## Consent

Written informed consent was obtained from the patient for publication of this case report and any accompanying images. A copy of the written consent is available for review by the Editor‐in‐Chief of this journal.

## Conflicts of Interest

The authors declare no conflicts of interest.

## Data Availability

The data that support the findings of this study are available on request from the corresponding author. The data are not publicly available due to privacy or ethical restrictions.
